# A Comparative Study of Ex-Vivo Murine Pulmonary Mechanics Under Positive- and Negative-Pressure Ventilation

**DOI:** 10.1007/s10439-023-03380-1

**Published:** 2023-10-31

**Authors:** K. A. M. Quiros, T. M. Nelson, A. Ulu, E. C. Dominguez, T. A. Biddle, D. D. Lo, T. M. Nordgren, M. Eskandari

**Affiliations:** 1 Department of Mechanical Engineering, University of California Riverside, 900 University Ave., Riverside, CA 92506 USA; 2 Division of Biomedical Sciences, Riverside School of Medicine, University of California, Riverside, CA USA; 3 Environmental Toxicology Graduate Program, University of California, Riverside, CA USA; 4 School of Medicine, BREATHE Center, University of California, Riverside, CA USA; 5 Center for Health Disparities Research, University of California, Riverside, CA USA; 6 Department of Environmental and Radiological Health Sciences, Colorado State University, Fort Collins, CO USA; 7 Department of Bioengineering, University of California, Riverside, CA USA

**Keywords:** Mouse lung, Pulmonary mechanics, Compliance, Viscoelasticity, Hysteresis, Positive-pressure, Negative-pressure

## Abstract

Increased ventilator use during the COVID-19 pandemic resurrected persistent questions regarding mechanical ventilation including the difference between physiological and artificial breathing induced by ventilators (i.e., positive- versus negative-pressure ventilation, PPV vs NPV). To address this controversy, we compare murine specimens subjected to PPV and NPV in ex vivo quasi-static loading and quantify pulmonary mechanics via measures of quasi-static and dynamic compliances, transpulmonary pressure, and energetics when varying inflation frequency and volume. Each investigated mechanical parameter yields instance(s) of significant variability between ventilation modes. Most notably, inflation compliance, percent relaxation, and peak pressure are found to be consistently dependent on the ventilation mode. Maximum inflation volume and frequency note varied dependencies contingent on the ventilation mode. Contradictory to limited previous clinical investigations of oxygenation and end-inspiratory measures, the mechanics-focused comprehensive findings presented here indicate lung properties are dependent on loading mode, and importantly, these dependencies differ between smaller versus larger mammalian species despite identical custom-designed PPV/NPV ventilator usage. Results indicate that past contradictory findings regarding ventilation mode comparisons in the field may be linked to the chosen animal model. Understanding the differing fundamental mechanics between PPV and NPV may provide insights for improving ventilation strategies and design to prevent associated lung injuries.

## Introduction

Uncovering differences between ventilating using positive- and negative-pressure has been a long-standing mission extending to the polio pandemic and “iron lung” [25]. Reminiscent of the polio pandemic, the COVID-19 pandemic and predicted urgency to ventilate patients caused a ventilator supply deficit, pushing the ingenuity and involvement of various companies (i.e. GM, Dyson, and Tesla) in ventilator design and manufacturing [2, 11, 16, 18]. Optimal settings for modern mechanical ventilators are vital [76], but the most reoccurring concern is whether to artificially ventilate with positive- or negative-pressure [2].

The two ventilation modes differ by how the transpulmonary pressure gradient is stimulated: in physiological breathing, the contracting diaphragm is responsible for inducing the pressure gradient; this can be mimicked by applying a negative-pressure (NPV) to the chest [83]. In positive-pressure ventilation (PPV), the gradient is implemented by forcing air through the trachea, directly expanding the lungs. Modern mechanical ventilators, for both invasive and emerging non-invasive ventilation, utilize PPV [19, 44]. Despite the demonstrated oxygenation and hemodynamic benefits, coupled with shorter periods of support associated with NPV [20, 33, 75], on top of the harmful side effects of short and long term PPV (including ventilator induced lung injury (VILI), pneumonia, diaphragm atrophy, and brain injury), PPV remains the main form of ventilation [4, 48, 57, 62, 67, 74]. Preference for PPV stems from cost-effective, compact equipment design, which is more feasible for clinical settings compared to more cumbersome negative-pressure devices that restrict patient access and care. Additionally, PPV allows for ventilation despite airway obstruction unlike NPV devices [2].

Previous studies of PPV versus NPV focused on clinical parameters, such as induced trauma, oxygenation, and blood flow [1, 8, 12, 33, 65]. While these end-result clinical factors are important for patient comfort and survival, their exploration has yielded contradictory results [9, 27, 33, 65] and perhaps a mechanics-focused approach to PPV versus NPV can provide complementary insights to clinical concerns, inform and optimize ventilator use and design to prevent damage, while adding to the experimental basis for informed and beneficial computer models [31, 42, 49, 63]. Mechanical comparisons of PPV and NPV previously required the use of multiple ventilators creating confounding factors stemming from mismatched ventilation strategies and different animal sets being used for each ventilation mode [1]. To address this concern, a new subset of custom-designed ventilation systems, to which ours belongs, allows testing of the same specimen in PPV and NPV to isolate the influence of the loading mechanism [24, 27, 52]. These systems are ex vivo and replicate PPV or NPV by pushing air into the lung or removing air from the air-tight tank enclosure respectively [24, 27]. Additionally, in contrast to many of these new systems, which are pressure-controlled, our device is volume-controlled allowing the insightful viscoelastic characterization of stress relaxation analyses [66, 71, 79].

Recent mechanics-focused (e.g., surface strain, elastance, peak-inspiratory measures) examinations have unearthed both ventilation mode dependencies and non-dependencies [24, 73]. Large and small mammalian species mechanics investigations (murine, ovine, and porcine) found equivalent end-inflation pressure and volume measurements whether testing with pressure- or volume-controlled apparatuses [24, 27, 73]. However, other mechanical measures in large mammalian species, such as resistance and elastance, yielded divergent results between modes; specifically, ovine lungs experienced lower lung resistance and elastance in NPV compared to PPV and noted varying levels of expansion of distal airways between the two modes [24]. Similarly, a porcine study observed ventilation modes dependencies via diverse surface strain tendencies between PPV and NPV [73]. Investigation of PPV/NPV dependencies in porcine specimens also revealed volume dependencies of compliance measures differ between PPV and NPV, demonstrating complex interdependencies regarding ventilation modes [73, 40].

Small-scale investigations of PPV versus NPV mechanics of murine lung mechanics have been pivotal in addressing the connection between oxygenation and fundamental pressure–volume (PV) mechanics [27]; however, these methods were limited to end-inspiratory and waveform mechanics. Moreover, the laboratory mouse has assisted in pulmonary research via the study of important questions ranging from lung cancer treatments to the basis of biological agents responsible for lung injury [26, 42]; the translational potential of exploring murine lung mechanics may offer advantageous insights to the root causes of greater levels of VILI [13, 33, 65] and to uncover anticipated disparate mechanical trends, given observed loading mechanism dependencies in larger specimens. As such, this murine study seeks to explore a comprehensive set of elastic and energetic measures over an expansive span of volumes and frequencies under both ventilation modes to enable insights regarding alveolar recruitment and viscoelastic behaviors lacking from the current literature [30, 80, 22].

## Materials and Methods

### Animals

C57BL/6J mice (*n* = 5, male, 8–12 weeks, 31.3 ± 4.5 g) were purchased through Jackson Laboratory (Bar Harbor, ME, USA), housed and monitored for 21 weeks under a 12:12 h light-dark cycle with standard mouse chow diet and drinking water ad-libitum. During which time, mice intranasally received 1× phosphate buffered saline (PBS) thrice weekly to serve as a control group in a larger study. Sacrifice was completed via isoflurane overdose. The trachea was cannulated (20-gauge) and the heart-lung bloc removed. An effort was made to minimize injury during this process, although the action of organ removal is inherently disruptive. Air was introduced into the lung (0.5 ml) to prevent atelectasis during transport. All experiments and procedures were approved by the University of California Riverside Institutional Animal Care and Use Committee (IACUC; protocol #20200014; some specimens were also utilized in Quiros et al. [64]).

### Positive- and Negative-Pressure Ventilation

PV testing occurred approximately three hours postmortem, using our validated custom-designed ventilator [70]. Briefly, PPV was accomplished by pushing an applied volume of air into the lungs through the secured cannula via tubing connected to a piston actuator. NPV was conducted by removing air from the tank to induce sub-atmospheric pressure. During the inflation-deflation cycle, real-time measurements of lung volume and transpulmonary pressure were collected. It is important to note the distinction between applied volume, which is delivered to the specimen via piston actuation, compared to our capability to measure compressed air lung volume directly via the secondary response piston [70]. The volume data presented here pertains to the more relevant behavior of the lung volume resulting from known applied volumes.

**Fig. 1:**
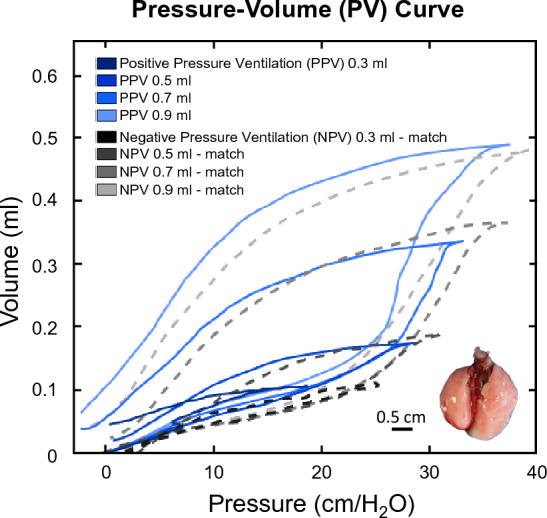
PV curves at matched volumes for PPV (blue, solid) and NPV (grey, dashed)

To evaluate various inflation volumes in PPV and NPV, four applied volumes were used under PPV (0.3, 0.5, 0.7, 0.9 ml) in ascending order. To comparably assess lung expansion between PPV/NPV, the applied volume under NPV was selected after PPV was conducted such that the resulting maximum recorded lung volumes between PPV and NPV were matched within ± 10% (Fig. 1). Preliminary studies showed the ordering sequence of PPV and NPV tests did not influence the tissue behavior, in agreement with recent studies [24]. Additionally, to quasi-statically minimize air flow resistance and investigate frequency trends, three cycling frequencies (5, 10, 20 breaths per minute, BPM) were tested [6, 39, 46].

For each volume and frequency tested, specimens’ volume history and consistent datum states were established by first applying a preload of 5 cmH_2_O, then preconditioning with three ventilation cycles [14, 64, 70, 80]. Subsequently, a fourth inflation-deflation cycle was implemented for analysis, followed by a fifth inflation and hold (24 seconds) to assess viscoelastic effects [30, 70]. All cycles were inflated to the tested peak volume and frequency.

### Calculations

As PV curves were collected under quasi-static conditions, it is assumed that peak pressure and plateau pressure are equal, thus quasi-static compliance was calculated as the ratio of maximum lung volume to peak pressure [3, 78]. Four measurements of dynamic compliance (as established in the literature [80]) were calculated via automated linear regression analysis over a range of data points such that *R*^2^ ≥ 0.90 at four regions of the breathing cycle using MATLAB (MathWorks Inc., Natick, MA, USA) as previously described and subsequentially confirmed [64, 80]: briefly, starting compliance (*C*_start_) and inflation compliance (*C*_inf_), as shown in Fig. 3A, were respectively calculated as the initial slope at the start of inflation and as the slope after the knee of the curve in the most compliant region (when visible) [80]. Additionally, top compliance (*C*_top_) and deflation compliance (*C*_def_), were respectively calculated as the slope at initial deflation and the slope of the curve at the end of deflation as shown in Fig. 4A (i.e., before and after the knee of the deflation curve) [64, 80]. Peak pressure was recorded at the end-inspiration. The viscoelastic measurement of percent relaxation, Fig. 6, was calculated as the percentage drop of pressure over the first 24 seconds to sufficiently measure the replicated response of a natural mouse sigh [84]. The energetic measure of hysteresis was calculated as the area enclosed by the PV curve and normalized to assess energy loss accounting for the increasing loop size for 0.5–0.9 ml (Fig. 7) [17, 53].

### Statistical Analysis

Results of all ten parameters are reported in Table 1 as mean ± standard deviation. Circles on bar graphs indicate individual specimen values. Black significance brackets demonstrate statistical differences between PPV and NPV. Blue and grey significance brackets indicate significance within PPV and NPV measurements, respectively, across volumes and frequencies. Statistical analysis was completed using GraphPad Prism 9 (Version 9.1.0, GraphPad Software, San Diego, CA, USA). Nonparametric analyses utilized to account for the small sample size. To compare volume and frequency variables, Friedman’s Test was utilized with post hoc analysis completed via Dunn’s Test [7]; comparison of PPV and NPV, was completed using Wilcoxon signed-rank test. As established in the literature, by virtue of the nature of Wilcoxon signed-rank test on small sample sizes, the minimum obtainable significance value of *p* = 0.0625 was used as the significance threshold [54, 55].

**Table 1:**
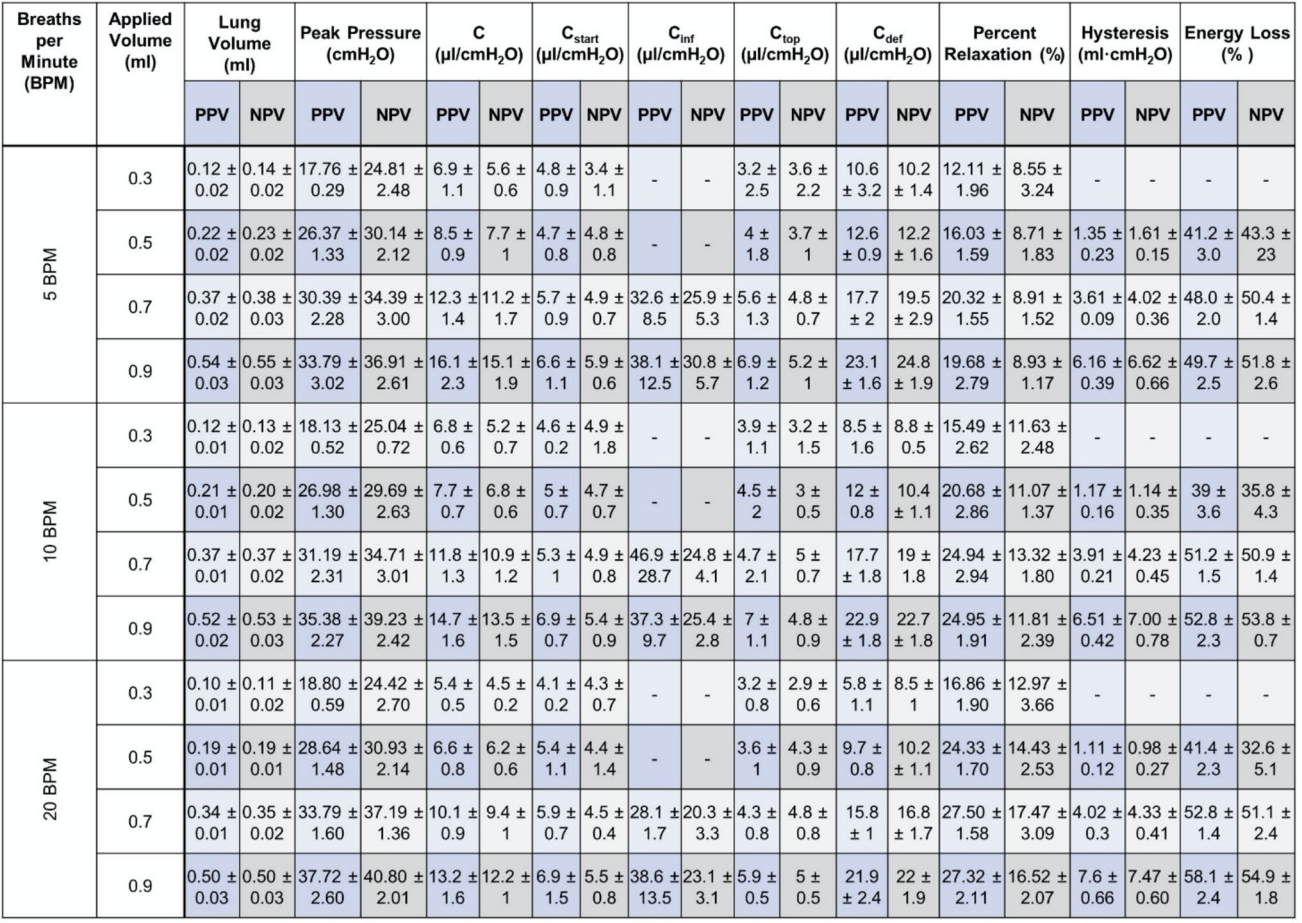
Mean ± SD of lung volume, peak pressure, quasi-static compliance, starting compliance, inflation compliance, top compliance, deflation compliance, percent relaxation, hysteresis, and energy loss in PPV and NPV for applied volumes 0.3, 0.5, 0.7, 0.9 ml at 5, 10, and 20 BPM

## Results

### Compliance

**Fig. 2:**
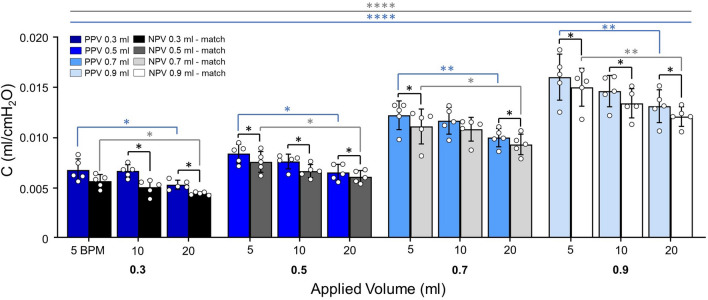
Quasi-static compliance was lower in NPV compared to PPV at most test sequences. Increased frequency decreased C for both ventilation modes. Increasing volume increased compliance for both modes indicated by Friedman’s test (*p* < 0.0001****)

Figure 2 shows NPV resulted in lower quasi-static compliance than PPV: except at 0.3 ml for 5 BPM and 0.7 ml for 10 BPM. For both PPV and NPV, increasing volume was found to increase quasi-static compliance (Friedman *p* < 0.0001, Fig. 2). Under PPV, increasing volume from 0.3 to 0.9 ml increased compliance at all frequencies, and increasing volume from 0.5 to 0.9 ml at 5 BPM increased compliance. Similarly, under NPV, compliance increased as volume increased from 0.3 to 0.9 ml at all frequencies. Increased frequency (5 to 20 BPM) decreased compliance in both ventilation modes at all volumes.

### Inflation Compliances

**Fig. 3:**
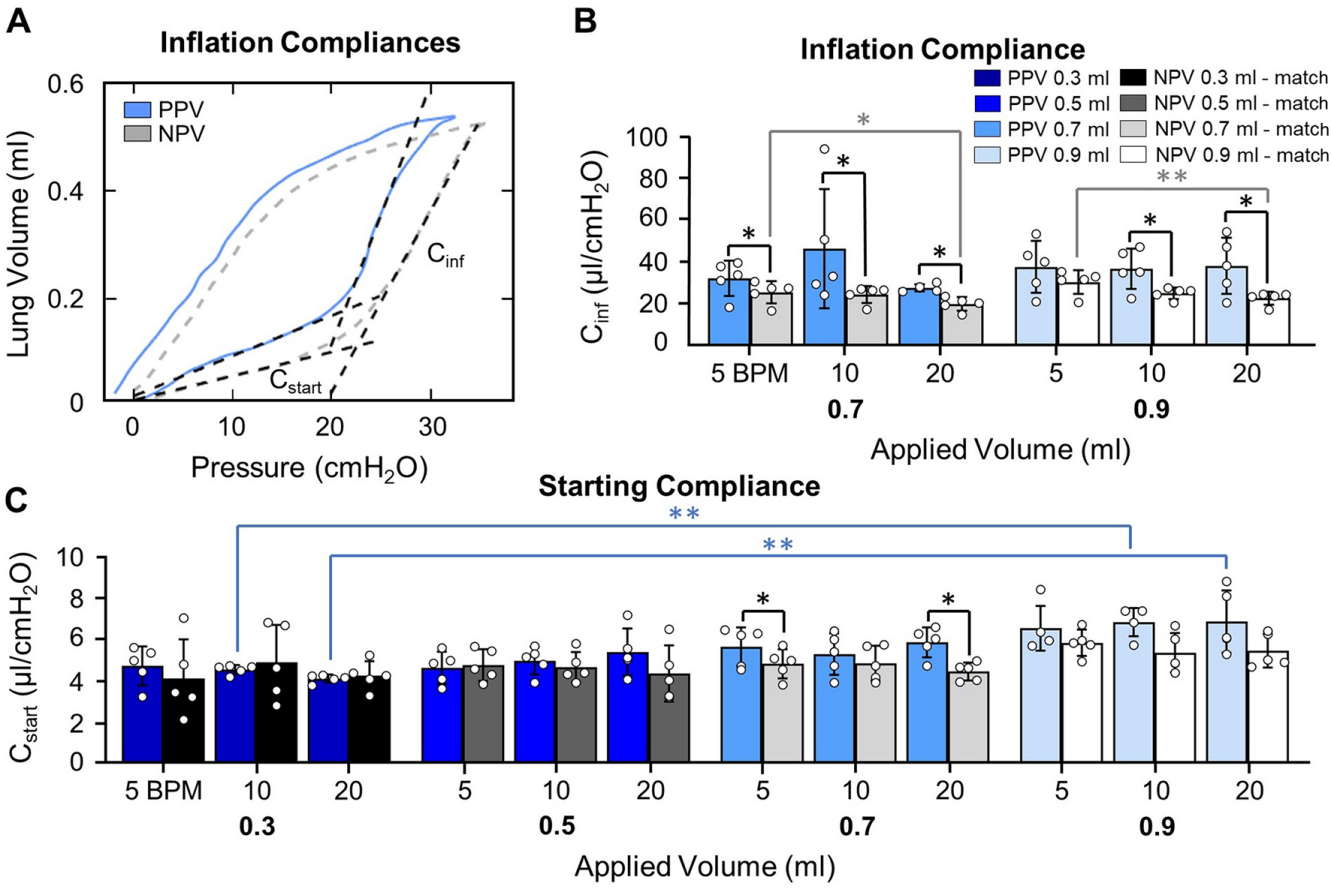
**A** PV curves demonstrating *C*_start_ and *C*_inf_. **B** *C*_inf_ was dependent on ventilation mode at most test sequences. Increased frequency decreased *C*_inf_ for NPV. **C** *C*_start_ was lower under NPV compared to PPV at 0.7 ml for 5 and 20 BPM. Increased volume increased *C*_start_ in PPV. Tangents are extended slightly past fit region for enhanced visibility

Analysis of compliance slopes in Fig. 3A, demonstrates *C*_inf_ was lower under NPV versus PPV; except at 0.9 ml for 5 BPM (Fig. 3B). *C*_inf_ was not volume dependent under either ventilation mode. Increased frequency (5 to 20 BPM) decreased *C*_inf_ under NPV at 0.7 and 0.9 ml.

Figure 3C shows *C*_start_ was lower under NPV compared to PPV at 0.7 ml for 5 and 20 BPM. Under PPV, *C*_start_ increased when volume increased from 0.3 to 0.9 ml at 10 and 20 BPM. *C*_start_ was not frequency dependent.

### Deflation Compliances

**Fig. 4:**
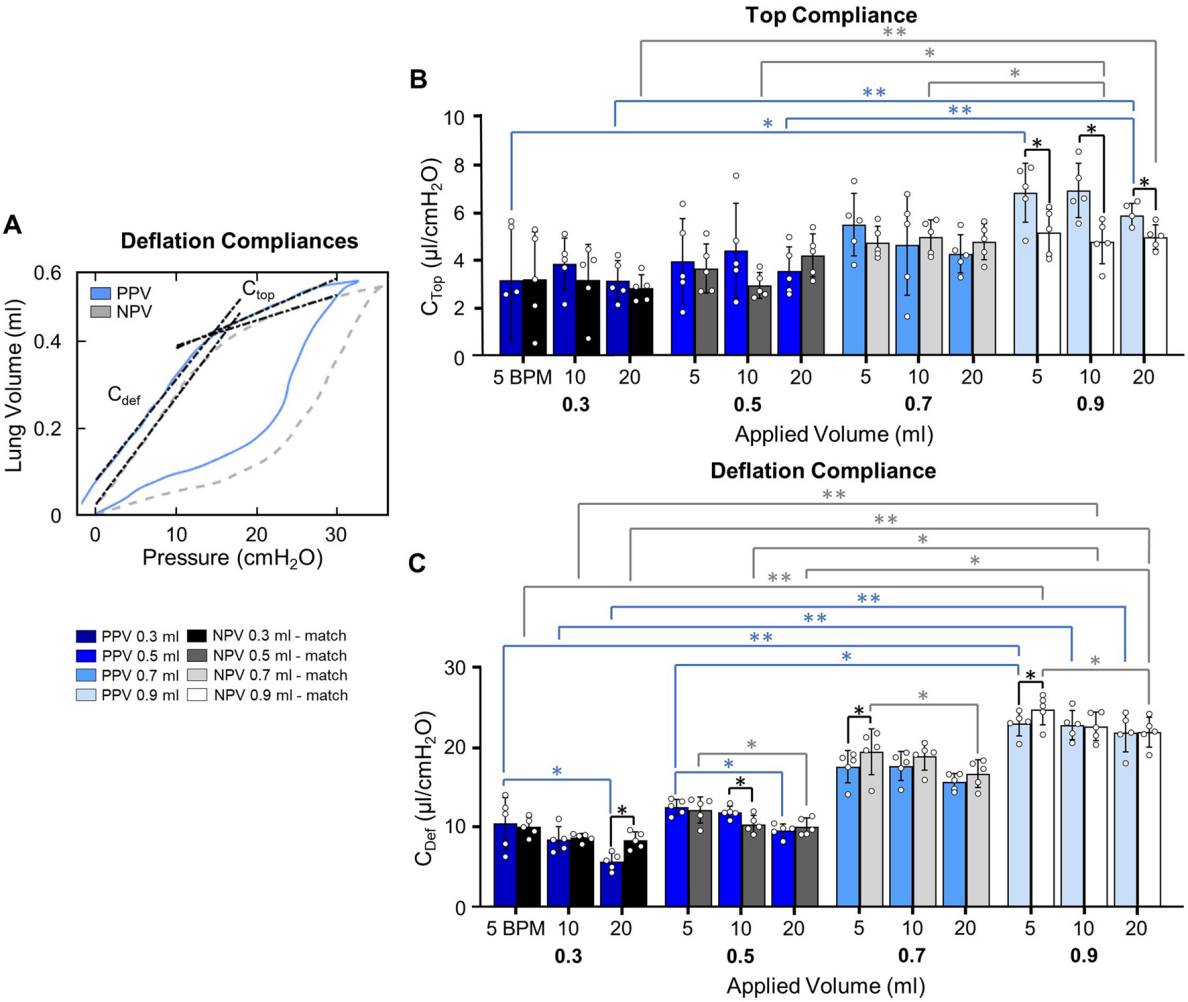
**A** PV curves demonstrating *C*_top_ and *C*_def_. **B** *C*_top_ was lower under NPV compared to PPV at 0.9 ml. *C*_top_ increased with increased volume in PPV and NPV, but was uninfluenced by BPM. **C** *C*_def_ was lower under PPV compared to NPV at a single BPM for each volume. *C*_def_ increased with increased volume under both modes and decreased with increased frequency. Tangents are extended slightly past fit region for enhanced visualization

Analysis of slopes in Fig. 4A, demonstrates *C*_top_ was lower in NPV compared to PPV at 0.9 ml (Fig. 4B). *C*_top_ increased with increased volume: For PPV, *C*_top_ increased when volume increased from 0.3 to 0.9 ml at 5 and 20 BPM, and from 0.5 to 0.9 ml at 20 BPM. Comparably, under NPV, *C*_top_ increased when volume increased from 0.3 to 0.9 ml at 20 BPM, from 0.5 to 0.9 ml at 10 BPM, and from 0.7 to 0.9 ml at 10 BPM.

Compared to NPV, *C*_def_ was lower for PPV at 5 BPM for 0.7 and 0.9 ml, at 10 BPM for 0.5 ml, and at 20 BPM for 0.3 ml (Fig. 4C). For PPV and NPV, increasing volume from 0.3 to 0.9 ml increased *C*_def_. Additionally, increasing from 0.5 to 0.9 ml increased *C*_def_ at 5 BPM for PPV, and at both 10 and 20 BPM for NPV. *C*_def_ decreased with increasing frequency: PPV was frequency dependent at 0.3 and 0.5 ml when frequency increased from 5 to 20 BPM, and NPV was dependent at 0.5, 0.7, and 0.9 ml for the transition from 5 to 20 BPM.

### Peak Pressure

**Fig. 5:**
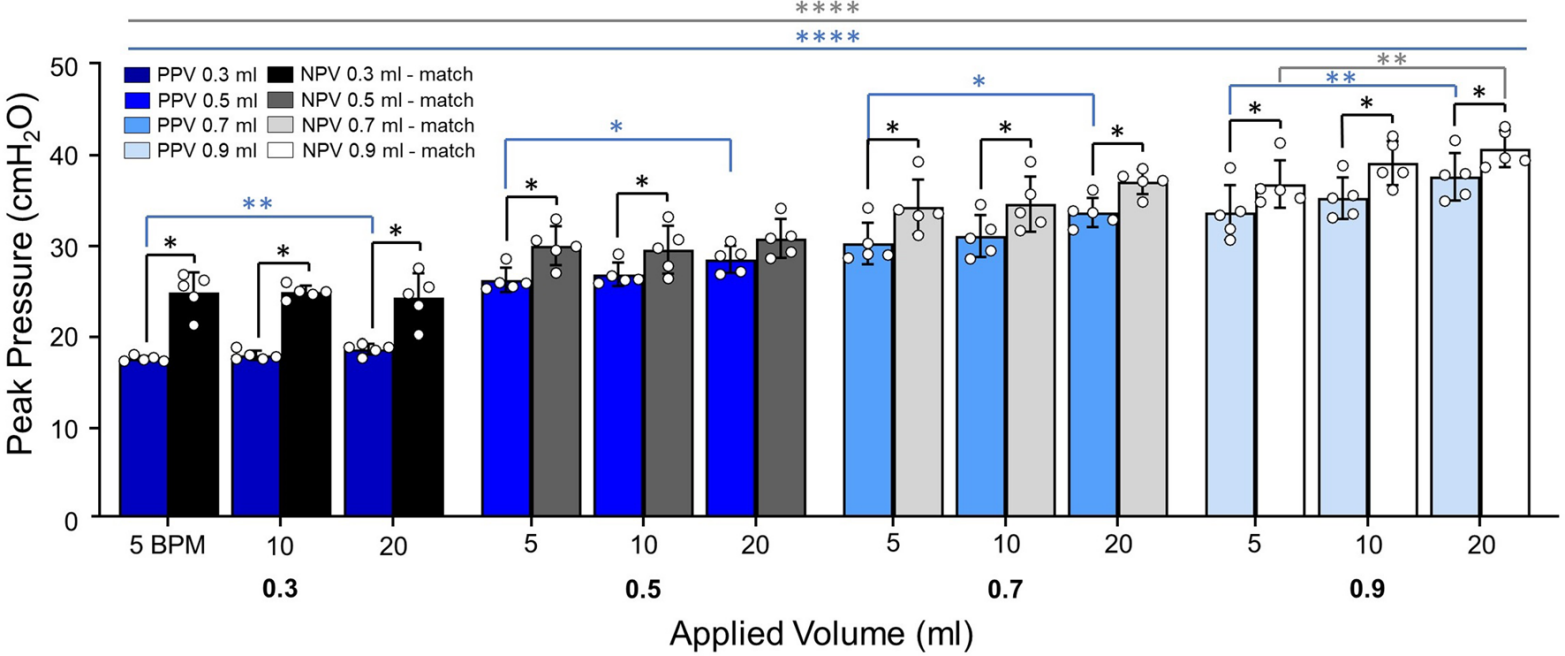
Peak pressure was lower in PPV compared to NPV at most test sequences. Increased BPM increased peak pressure during PPV and similarly affected NPV at a single transition. Increased volume increased peak pressure figure for both ventilation modes

Figure 5 shows peak pressure was lower under PPV compared to NPV; except for 0.5 ml at 20 BPM. Peak pressure increased with increasing volume (Friedman *p* < 0.0001). For PPV, peak pressure was frequency dependent at all volumes from 5 to 20 BPM. At lower volumes no peak pressure frequency dependence was found under NPV, however, at 0.9 ml peak pressure was frequency dependent.

### Percent Relaxation

**Fig. 6:**
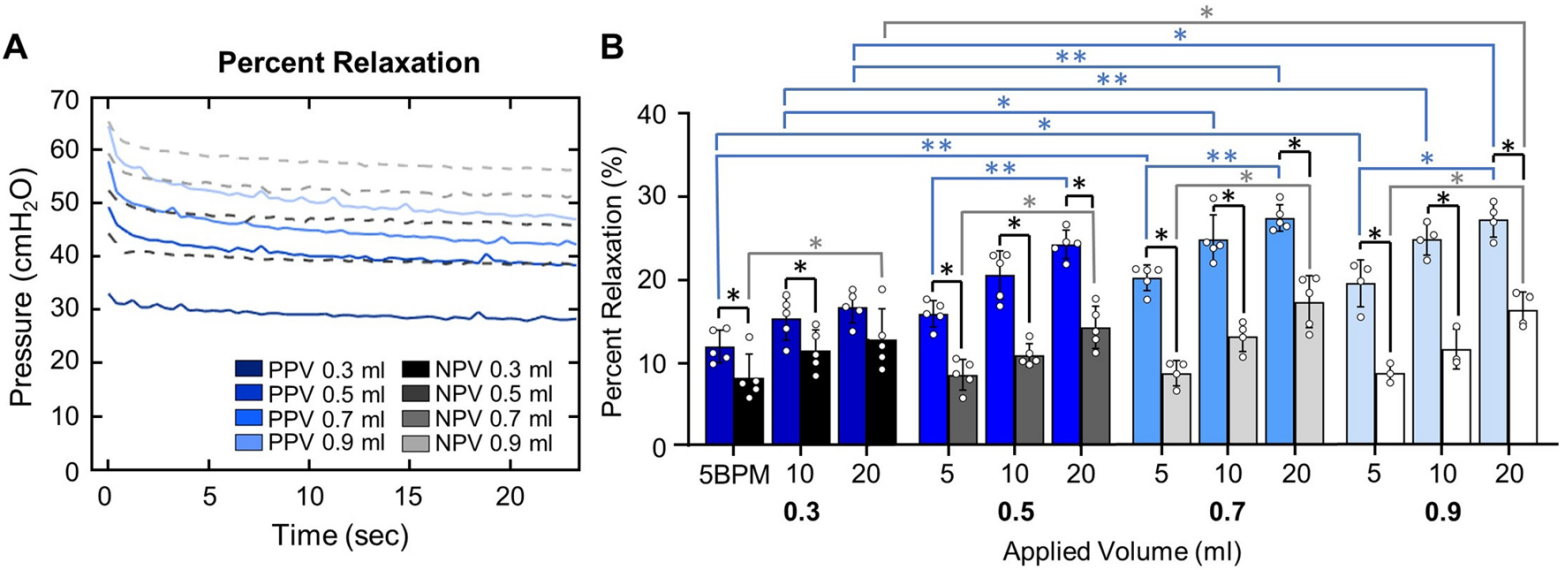
**A** Comparison of viscoelastic response in PPV and NPV over 24 s hold (PPV, solid blue; NPV, dashed grey). **B** Percent relaxation was lower under NPV compared to PPV at most test sequences. Increased volume increased percent relaxation under PPV and similarly under NPV increased volume increased percent relaxation across a single volume transition. Increasing frequency increased percent relaxation for PPV and NPV

Percent relaxation (Fig. 6A) was lower under NPV compared to PPV, except for 0.3 ml at 20 BPM (Fig. 6B). Percent relaxation was volume dependent almost exclusively in PPV, where viscoelastic relaxation increased with increasing volume from 0.3 to 0.9 ml and from 0.3 to 0.7 ml. In contrast, under NPV, one instance of volume dependence occurred (0.3 to 0.9 ml at 20 BPM). Relaxation was frequency dependent for both ventilation modes: increasing with frequency for PPV at 0.5, 0.7, and 0.9 ml and under NPV at all volumes.

### Energetics

**Fig. 7:**
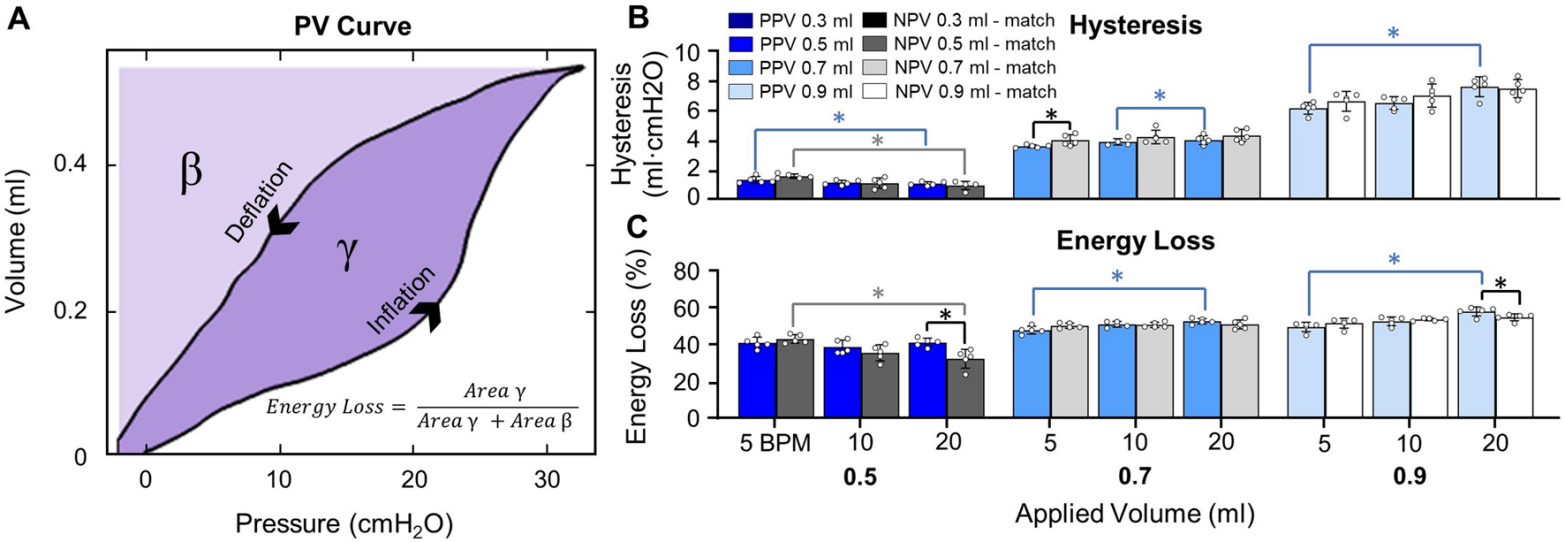
**A** Hysteresis and energy loss calculation as $\gamma$ and $\frac{\gamma }{\gamma +\beta }$ respectively. **B** Hysteresis was lower under PPV compared to NPV at 0.7 ml and 5 BPM. Under PPV, increased frequency decreased hysteresis at 0.5 ml but increased hysteresis at higher volumes. Under NPV, only 0.5 ml resulted in frequency dependence. **C** Energy loss was lower in NPV compared to PPV at 20 BPM for 0.5 and 0.9 ml. Increased BPM increased energy loss in PPV. Conversely, under NPV, increased BPM decreased energy loss at 0.5 ml

The energetics (Fig. 7A) had few instances of significance between ventilation modes; for hysteresis (Fig. 7B), only 0.5 ml at 5 BPM resulted in higher hysteresis under NPV compared to PPV. Lower energy loss (Fig. 7C) under NPV, in comparison to PPV, occurred for 0.5 and 0.9 ml at 20 BPM. Both energetics frequency trends differed between ventilation modes at higher volumes. In PPV, hysteresis and energy loss increased with increasing frequency at 0.7 and 0.9 ml, but in NPV neither energetic measure was frequency dependent. Additionally, for both ventilation modes, hysteresis decreased with increasing frequencies from 5 to 20 BPM at 0.5 ml. This pattern remained after normalization in NPV (i.e., energy loss decreased between 5 and 20 BPM at 0.5 ml) but was not observed in PPV.

## Discussion

### Key Findings

In this study we examine the dependencies of elastic, energetic, and viscoelastic measures on loading mechanism, namely positive- versus negative- pressure ventilation. Interdependencies arising from varied inflation volume and frequencies under the two ventilation strategies are also investigated. Critical findings are as follows: (1) For all ten reported parameters, the values varied significantly between ventilation modes at select volume and frequency pairings. Furthermore, we note differing volume and frequency dependencies between loading mechanisms as follows: (2) Peak pressure is found to be dependent on cycling rate in only PPV. (3) Elastic measurements show starting compliance is affected by volume in PPV, and inflation compliance is frequency dependent in NPV. (4) Viscoelastic measures reveal differing dependencies: percent relaxation is almost exclusively affected by changing volume under PPV; hysteresis and energy loss are not intrinsically volume dependent but are influenced by frequency in PPV and NPV. Interestingly, volume and frequency interdependencies are noted under both loading modes; at low volumes, hysteresis and energy loss decrease with frequency for both ventilation modes but, at higher volumes, energetic measures increase with frequency in PPV only. In the following sections, we present these results in the context of the literature and investigate these trends, hypothesizing the reasons behind how these measures are connected to the underlying physiology.

### Inflation Compliances

*C*_start_ in this study was volume dependent under PPV; aeration and alveolar recruitment are potential causes of this single mode dependency. In previous larger mammalian studies, *C*_start_ was not volume dependent under PPV [73, 80, 82], but rather *C*_start_ was volume dependent when ventilated with NPV. The contrasting *C*_start_ trends in the porcine study exist despite near identical calculation methods, with similarly high confidence intervals (*R*^2^ > 0.90), and posited delayed recruitment in PPV was the main explanatory variable for the *C*_start_ trends [73]; as more alveoli are recruited during NPV with increasing volume, the stress on initially opened alveoli is eased, altering starting compliance. Applying that logic here implies the inverted significances for murine subjects means the reverse, where greater recruitment occurred during *C*_start_ in PPV.

However, alveolar recruitment does not occur in mice during *C*_start_, rather, alveoli expand under PPV but do not become more numerous [56]. The lack of recruitment during *C*_start_ is agreeable with the noted correlation between *C*_start_ and the level of tissue-aeration at end-expiration (atmospheric pressure) [32, 60]. The two ideas are directly correlated such that instead of new alveoli opening, the compliance is attributable to the number of opened alveoli at the start of inspiration and should therefore not be altered by further inflation. Neither notion, recruitment nor tissue-aeration, explain the PPV volume dependency seen here. However, when coupled with previous findings that the amount of aerated tissue at end-expiration is dependent on volume history [21], the dependency of initial tissue aeration may explain our noted volume dependency. Moreover, aerated tissue dependency on inflation history has been observed in a clinical study at high plateau pressures, where aeration at end-expiration increased with increasing peak inflation pressures for mechanically ventilated subjects [21]. While not assessed by Crotti et al., our observations suggest the trend persists at lower inflation pressures causing our observed volume dependencies. Together, these findings suggest aeration may be volume dependent under mechanical ventilation alone, causing differing trends between the two modes. It remains to be explored as to why this trend is reversed in larger specimens.

Additionally, previous comparisons of PPV and NPV mechanics demonstrated matched volume history led to matched pulmonary mechanics, and the percentage of aerated tissue at end-expiration was comparable between the two modes [27, 36]. Given this study’s use of the matched volume history establishment, and the presumed matched initial aeration, *C*_start_ should not vary between ventilation modes, as seen here, if it is strictly dependent on the amount of initial aerated tissue. This implies other potential contributing factors which result in differing volume dependencies and instances of statistically significant steeper slopes under PPV compared to NPV. As such, the ventilation mode discrepancies regarding *C*_start_ warrants further study.

Similarly, opposing trends between species occur for *C*_inf_. In porcine and ovine lungs, *C*_inf_ varied with inflation volume in PPV and NPV, and with pressure in PPV, respectively; whereas for murine lungs, neither ventilation mode demonstrated *C*_inf_ volume dependency [24, 73]. Recruitment may play an important role in the differing trends of *C*_inf_, given *C*_inf_’s link to recruitment [60], where the disparate lung structure of the three mammalian species is responsible for altered recruitment levels. Specifically, the marked increased collateral ventilation noted in murine lungs versus porcine lungs may be, in part, responsible for the differing trends [43, 61]. In the region of *C*_inf_, recruitment of alveoli via collateral ventilation is documented in the murine lung (i.e., increasing the regions available for inflation to accommodate larger volumes and prevent volume dependency) [56]. While collateral ventilation is not a prominent mode of air distribution in porcine specimens, it is demonstrated in ovine specimens. Therefore, collateral ventilation is unlikely the main cause for volume dependency trends given ovine and porcine specimens share dependencies, but murine and ovine subjects share secondary recruitment tactics [45].

### End-Inspiration Behavior

Comparing end-inspiratory pressure under matched volumes between PPV and NPV resulted in higher pressures under NPV. Increased peak pressure can be indicative of injury in the form of surfactant disruption, but as the same lung was tested for PPV and NPV, any surfactant contributions would be nearly, if not completely, identical between the two modes [81]. Airway resistance could additionally be responsible for the disparity but can be neglected as the quasi-static nature of the experiments results in a relatively small resistive contribution to overall pressure [6]. Investigation of peak pressure has yielded conflicting results in both quasi-static and non-quasi-static studies [24, 65, 73]. In contrast to our findings, ex vivo larger mammalian studies have reported matched peak volume and pressure behavior between modes [24, 61, 73]: an ovine study noted both ventilation modes resulted in matched maximum pressures, despite hypothesizing a differing interior pressure gradient may coincide with noted uneven peripheral airway distension between modes [24]. It was suggested that this gradient occurs too deep in the lung to detect [24]. However, murine lungs are substantially smaller, potentially allowing the divergent peripheral airway opening and coupled pressure gradient to be closer to the trachea, a potential cause for the observed disparate pressures.

On the other hand, a previous murine investigation found transpulmonary pressure had no ventilation mode dependency [27]. The matched end-inspiratory mechanics from Engelberts et al. disagree with our diverse peak pressures, despite comparable testing volumes (Engelberts: ~ 0.22–0.45 ml; this study: 0.3, 0.5 ml) [27]. Engelberts’ ex vivo study was performed in situ, and it is not obvious if the contributing effects from the chest cavity and surrounding tissues could explain disparate trends [38]. However, differing peak-inspiratory behavior from PPV and NPV have been noted in in vivo clinical studies suggesting the presence of the ribcage alone does not eliminate divergent pressure responses [65]. While the volumes were comparable, the cycling frequencies were not (Engelberts: 90 bpm; this study: 5–20 bpm). Indicating the contribution of resistance in Engelberts’ study may be causing the discrepancy, as resistance is comparably negligible in this quasi-static study [27].

Additionally, the frequency trends of peak pressure differ between modes. For 0.3–0.7 ml, peak pressure increases with increasing frequency in only PPV. At 0.9 ml, peak pressure varied with frequency under both modes. The NPV dependency has not previously been investigated, and the differing dependencies between the two modes merits further study.

### Viscoelastic Response

Despite testing the same specimen under both ventilation modes—reducing inter-specimen variability by comparing each lung’s behavior to itself under either PPV or NPV—we demonstrate lower stress relaxation in NPV compared to PPV similar to porcine lungs [73]. While stress relaxation is knowingly influenced by a variety of dependencies (e.g. temperature, volume, and rate), only recently was the loading mode discovered to influence this metric, and this study concurs [64, 69, 73]. Given the specimens are shared between modes in this study, the contributing effects of tissue and surfactant [5, 30, 47] are consistent and, therefore are unlikely the source of variability. A new theory posits PPV/NPV differences are attributable to alveoli loading acting as thick-walled vessels instead of the traditionally modeled thin-walled systems [73]. Another contribution to lower relaxation in NPV may be the increased openness of peripheral airways in NPV [24, 29]; given the lower stress relaxation of the small airway tissues, the larger contribution of small airways at end-inflation may result in altered relaxation contributions and conflicting end-results between modes [24, 30].

Similarly, and in agreement with Sattari and colleagues, we found differing stress relaxation volume dependencies between the two modes [73]. PPV was more dependent on volume, with only one point of significance in NPV. The volume and frequency dependencies of percent relaxation have previously been investigated under solely PPV with differing findings [64, 69]. In this study, we note that PPV relaxation increased with increasing frequency while another conflicting study noted opposing trends where relaxation decreased with increasing flow rates [23]. However, both studies conclude viscoelastic relaxation increases with increasing volume in the quasi-static realm [23]. This finding is supported by prior investigations of viscoelasticity under dynamic conditions which also demonstrate viscoelastic volume dependence [68]. Given the demonstrated volume and frequency dependencies of the viscoelastic contributions of pulmonary tissue, the influence of viscoelasticity on PV curves should not be discounted as a potential contribution to the frequency and volume dependencies noted within this study for all mechanics [68, 79, 86].

### Energetics

We find interdependencies between ventilation mode and frequency for energetic measures are altered at larger volumes despite duplicate specimen testing resulting in identic energetic contributions of surface tension and tissue [85]. At lower volumes, the frequency dependencies of hysteresis and energy loss trends were comparable between the two modes. At higher volumes, PPV frequency dependence reversed and NPV was unaffected. In PPV, the frequency dependence of hysteresis has been noted in mice, dog, and cat lungs [34, 37, 64], and the noted volume and frequency interdependency under PPV have also been demonstrated [34]. This PPV interdependency has been noted for other lung mechanics, such as local surface strains, and may indicate differing frequency dependences among tissues in the extracellular matrix given the modified mechanical contributions of tissues (e.g. collagen) across volumes especially considering the strain dependence of airway and surface tissues [47–52, 58, 72, 77].

However, in NPV, while frequency dependencies on work of breathing have been noted [59], we find this joined volume and frequency interdependency of energetic measures under quasi-static conditions for the first time. Investigation in clinical applications have revealed the interconnection between volume and frequency on the effects of gas exchange; mechanical negative-pressure devices (i.e., Hayek oscillator) operate most efficiently at higher volumes and lower frequencies [10, 15, 35]. For clinical observations, varying loading parameters outside of the quasi-static regime introduces complex flows (i.e., pendelluft) caused by alveolar pressure gradients that alter mechanics [28]. Under the inflation protocols investigated here, disruptive flow patterns should not be present, but differing recruitment levels may alter frequency dependence due to recruitment potentially varying across ventilation modes [10, 73, 86].

### Limitations

This study seeks to characterize organ mechanics with ex vivo testing, and therefore, the contributions of the chest wall are not analyzed despite their importance in respiratory mechanics; however, the influence of the soft chest wall on murine mechanics is less substantial [38, 41]. Additionally, lungs utilized here were not degassed in an effort to conduct a near physiological assessment of the organ. Not degassing allows for superior assessment compared to degassed lungs which are inherently damaged by the collapse of airways and alveoli. Although the measurement sequence was not randomized, the semi-crossover design of this study allowed paired comparison of PPV and NPV while accounting for ex vivo testing conditions (e.g., chest wall, surfactant disruption, etc.), given the same specimen was tested under both loading modes. While all tests were conducted under quasi-static conditions to isolate compliance behavior, making flow resistance negligible, the study of volume and frequency dependencies unavoidably results in comparison of different flow rates which alters knowingly negligible flow resistive contributions of PV curves. Additionally, due to the delicate nature of soft tissue testing, specifically the intricate organ removal process, damage and leaks were unavoidable, resulting in injury and causing dozens of lungs to be dissected but limiting the successful acquisition of unpunctured specimens and thus the sample size.

## References

[1] Aboelnazar NS, Himmat S, Hatami S, White CW, Burhani MS, Dromparis P, Matsumura N, Tian G, Dyck JRB, Mengel M, Freed DH, Nagendran J (2018). Negative pressure ventilation decreases inflammation and lung edema during normothermic ex-vivo lung perfusion. J. Heart Lung Transplant..

[2] Abughanam N, Gaben SSM, Chowdhury MEH, Khandakar A (2021). Investigating the effect of materials and structures for negative pressure ventilators suitable for pandemic situation. Emergent Mater..

[3] Albaiceta GM, Taboada F, Parra D, Blanco A, Escudero D, Otero J (2003). Differences in the deflation limb of the pressure–volume curves in acute respiratory distress syndrome from pulmonary and extrapulmonary origin. Intensive Care Med..

[4] Bassi TG, Rohrs EC, Fernandez KC, Ornowska M, Nicholas M, Gani M, Evans D, Reynolds SC (2021). Brain injury after 50 h of lung-protective mechanical ventilation in a preclinical model. Sci. Rep..

[5] Bates JH, Maksym GN, Navajas D, Suki B (1994). Lung tissue rheology and 1/f noise. Ann. Biomed. Eng..

[6] Bayliss LE, Robertson GW (1939). The Visco-elastic properties of the lungs. Q. J. Exp. Physiol. Cogn. Med. Sci..

[7] Becher T, Wendler A, Eimer C, Weiler N, Frerichs I (2019). Changes in electrical impedance tomography findings of ICU patients during rapid infusion of a fluid bolus: a prospective observational study. Am. J. Respir. Crit. Care Med..

[8] von Bethmann AN, Brasch F, Nüsing R, Vogt K, Volk HD, Müller K-M, Wendel A, Uhlig S (1998). Hyperventilation induces release of cytokines from perfused mouse lung. Am. J. Respir. Crit. Care Med..

[9] Bobba CM, Nelson K, Dumond C, Eren E, Black SM, Englert JA, Ghadiali SN, Whitson BA (2021). A novel negative pressure-flow waveform to ventilate lungs for normothermic ex vivo lung perfusion. ASAIO J..

[10] Bohn DJ, Miyasaka K, Marchak BE, Thompson WK, Froese AB, Bryan AC (1980). Ventilation by high-frequency oscillation. J. Appl. Physiol..

[11] Branson RD, Rodriquez D (2023). COVID-19 lessons learned: response to the anticipated ventilator shortage. Respir. Care.

[12] Bruells CS, Smuder AJ, Reiss LK, Hudson MB, Nelson WB, Wiggs MP, Sollanek KJ, Rossaint R, Uhlig S, Powers SK (2013). Negative pressure ventilation and positive pressure ventilation promote comparable levels of ventilator-induced diaphragmatic dysfunction in rats. Anesthesiology.

[13] Bryda EC (2013). The Mighty Mouse: the impact of rodents on advances in biomedical research. Mo. Med..

[14] Carew EO, Barber JE, Vesely I (2000). Role of preconditioning and recovery time in repeated testing of aortic valve tissues: validation through quasilinear viscoelastic theory. Ann. Biomed. Eng..

[15] Chang HK (1984). Mechanisms of gas transport during ventilation by high-frequency oscillation. J. Appl. Physiol..

[16] Chiang LE, Castro FA (2021). VEMERS UC: a clinically validated emergency mechanical ventilator for COVID-19 and postpandemic use in low resource communities. J. Med. Device.

[17] Chung, J., K. Lachapelle, E. Wener, R. Cartier, B. De Varennes, R. Fraser, and R. L. Leask. Energy loss, a novel biomechanical parameter, correlates with aortic aneurysm size and histopathologic findings. *J. Thorac. Cardiovasc. Surg.* 148:1082–1088; discussion 1088–1089, 2014. 10.1016/j.jtcvs.2014.06.021 25129601

[18] Cole JH, Hughey SB, Rector CH, Booth GJ (2020). A novel low-cost ventilator for use in a worldwide pandemic: the portsmouth ventilator. Crit. Care Explor..

[19] Corrado A, Gorini M (2002). Long-term negative pressure ventilation. Respir. Care Clin. N. Am..

[20] Corrado A, Gorini M, Ginanni R, Pelagatti C, Villella G, Buoncristiano U, Guidi F, Pagni E, Peris A, De Paola E (1998). Negative pressure ventilation versus conventional mechanical ventilation in the treatment of acute respiratory failure in COPD patients. Eur. Respir. J..

[21] Crotti S, Mascheroni D, Caironi P, Pelosi P, Ronzoni G, Mondino M, Marini JJ, Gattinoni L (2001). Recruitment and derecruitment during acute respiratory failure: a clinical study. Am. J. Respir. Crit. Care Med..

[22] Mariano, C. A., S. Samaneh, G. O. Ramirez, and M. Eskandari. Effects of tissue degradation by collagenase and elastase on the biaxial mechanics of porcine airways. *Respir. Res.* 24(1):105, 2023. 10.1186/s12931-023-02376-8 10.1186/s12931-023-02376-8 PMC10082978 37031200

[23] D’Angelo E, Calderini E, Torri G, Robatto FM, Bono D, Milic-Emili J (1989). Respiratory mechanics in anesthetized paralyzed humans: effects of flow, volume, and time. J. Appl. Physiol..

[24] Dong, S., L. Wang, P. Chitano, H. O. Coxson, D. M. Vasilescu, P. D. Paré, and C. Y. Seow. Lung resistance and elastance are different in ex vivo sheep lungs ventilated by positive and negative pressures. *Am. J. Physiol. Lung Cell. Mol. Physiol.*, 2022. 10.1152/ajplung.00464.2021. 10.1152/ajplung.00464.2021 35272489

[25] Drinker PA, McKhann CF (1986). The iron lung: first practical means of respiratory support. JAMA.

[26] Eldh T, Heinzelmann F, Velalakan A, Budach W, Belka C, Jendrossek V (2012). Radiation-induced changes in breathing frequency and lung histology of C57BL/6J mice are time- and dose-dependent. Strahlenther. Onkol..

[27] Engelberts D, Malhotra A, Butler JP, Topulos GP, Loring SH, Kavanagh BP (2012). Relative effects of negative versus positive pressure ventilation depend on applied conditions. Intensive Care Med..

[28] Enokidani Y, Uchiyama A, Yoshida T, Abe R, Yamashita T, Koyama Y, Fujino Y (2021). Effects of ventilatory settings on Pendelluft phenomenon during mechanical ventilation. Respir. Care.

[29] Eskandari, M. Reply to Dong et al. *Am. J. Respir. Crit. Care Med.* 207(6):800–801 2023. 10.1164/rccm.202211-2108LE 10.1164/rccm.202211-2108LE PMC10037484 36395487

[30] Eskandari M, Arvayo AL, Levenston ME (2018). Mechanical properties of the airway tree: heterogeneous and anisotropic pseudoelastic and viscoelastic tissue responses. J. Appl. Physiol..

[31] Eskandari M, Nordgren TM, O’Connell GD (2019). Mechanics of pulmonary airways: linking structure to function through constitutive modeling, biochemistry, and histology. Acta Biomater..

[32] Gattinoni L, Pesenti A, Avalli L, Rossi F, Bombino M (1987). Pressure–volume curve of total respiratory system in acute respiratory failure. Computed tomographic scan study. Am. Rev. Respir. Dis..

[33] Grasso F, Engelberts D, Helm E, Frndova H, Jarvis S, Talakoub O, McKerlie C, Babyn P, Post M, Kavanagh BP (2008). Negative-pressure ventilation: better oxygenation and less lung injury. Am. J. Respir. Crit. Care Med..

[34] Grotberg JB, Davis SH (1980). Frequency dependence of pressure–volume loops in isolated dog lobes. J. Biomech..

[35] Hardinge FM, Davies RJ, Stradling JR (1995). Effects of short term high frequency negative pressure ventilation on gas exchange using the Hayek oscillator in normal subjects. Thorax.

[36] Helm E, Talakoub O, Grasso F, Engelberts D, Alirezaie J, Kavanagh BP, Babyn P (2009). Use of dynamic CT in acute respiratory distress syndrome (ARDS) with comparison of positive and negative pressure ventilation. Eur. Radiol..

[37] Hildebrandt J (1969). Dynamic properties of air-filled excised cat lung determined by liquid plethysmograph. J. Appl. Physiol..

[38] Hirai T, McKeown KA, Gomes RF, Bates JH (1999). Effects of lung volume on lung and chest wall mechanics in rats. J. Appl. Physiol..

[39] Huang K, Rabold R, Schofield B, Mitzner W, Tankersley CG (2007). Age-dependent changes of airway and lung parenchyma in C57BL/6J mice. J. Appl. Physiol..

[40] Investigating the Mechanics of Positive- versus Negative-PressureVentilation

[41] Ito S, Ingenito EP, Brewer KK, Black LD, Parameswaran H, Lutchen KR, Suki B (2005). Mechanics, nonlinearity, and failure strength of lung tissue in a mouse model of emphysema: possible role of collagen remodeling. J. Appl. Physiol..

[42] Jaecklin, T., D. Engelberts, G. Otulakowski, H. O’Brodovich, M. Post, and B. P. Kavanagh. Lung-derived soluble mediators are pathogenic in ventilator-induced lung injury. *Am. J. Physiol. Lung Cell. Mol. Physiol.* 300:648–658, 2011. 10.1152/ajplung.00305.2010 21239530

[43] Judge EP, Hughes JML, Egan JJ, Maguire M, Molloy EL, O’Dea S (2014). Anatomy and bronchoscopy of the porcine lung. A model for translational respiratory medicine. Am. J. Respir. Cell Mol. Biol..

[44] Katira BH (2019). Ventilator-induced lung injury: classic and novel concepts. Respir. Care.

[45] Kuriyama T, Wagner WW (1981). Collateral ventilation may protect against high-altitude pulmonary hypertension. J. Appl. Physiol..

[46] Limjunyawong N, Fallica J, Horton MR, Mitzner W (2015). Measurement of the pressure–volume curve in mouse lungs. J. Vis. Exp..

[47] Lorino AM, Harf A, Atlan G, Lorino H, Laurent D (1982). Role of surface tension and tissue in rat lung stress relaxation. Respir. Physiol..

[48] Luo J, Wang M-Y, Zhu H, Liang B-M, Liu D, Peng X-Y, Wang R-C, Li C-T, He C-Y, Liang Z-A (2014). Can non-invasive positive pressure ventilation prevent endotracheal intubation in acute lung injury/acute respiratory distress syndrome? A meta-analysis. Respirology.

[49] Maghsoudi-Ganjeh M, Mariano CA, Sattari S, Arora H, Eskandari M (2021). Developing a lung model in the age of COVID-19: a digital image correlation and inverse finite element analysis framework. Front. Bioeng. Biotechnol..

[50] Maghsoudi-Ganjeh M, Sattari S, Eskandari M (2021). Mechanical behavior of the airway wall in respiratory disease. Curr. Opin. Physiol..

[51] Mariano CA, Sattari S, Maghsoudi-Ganjeh M, Tartibi M, Lo DD, Eskandari M (2020). Novel mechanical strain characterization of ventilated ex vivo porcine and murine lung using digital image correlation. Front. Physiol..

[52] Mariano CA, Sattari S, Quiros KAM, Nelson TM, Eskandari M (2022). Examining lung mechanical strains as influenced by breathing volumes and rates using experimental digital image correlation. Respir. Res..

[53] Mead J, Whittenberger JL, Radford EP (1957). Surface tension as a factor in pulmonary volume–pressure hysteresis. J. Appl. Physiol..

[54] Meek GE, Ozgur C, Dunning K (2007). Comparison of the t vs. Wilcoxon signed-rank test for likert scale data and small samples. J. Mod. Appl. Stat. Methods.

[55] Muschter D, Geyer F, Bauer R, Ettl T, Schreml S, Haubner F (2018). A comparison of cell survival and heat shock protein expression after radiation in normal dermal fibroblasts, microvascular endothelial cells, and different head and neck squamous carcinoma cell lines. Clin. Oral Investig..

[56] Namati E, Thiesse J, de Ryk J, McLennan G (2008). Alveolar dynamics during respiration: are the pores of Kohn a pathway to recruitment?. Am. J. Respir. Cell Mol. Biol..

[57] Nelson TM, Quiros KAM, Dominguez EC, Ulu A, Nordgren TM, Eskandari M (2023). Diseased and healthy murine local lung strains evaluated using digital image correlation. Sci. Rep..

[58] Nelson TM, Quiros KAM, Mariano CA, Sattari S, Ulu A, Dominguez EC, Nordgren TM, Eskandari M (2022). Associating local strains to global pressure–volume mouse lung mechanics using digital image correlation. Physiol. Rep..

[59] Otis AB (1954). The work of breathing. Physiol. Rev..

[60] Pelosi P, Gattinoni L (2000). Respiratory mechanics in ARDS: a siren for physicians?. Intensive Care Med..

[61] Port CD, Ketels KV, Coffin DL, Kane P (1977). A comparative study of experimental and spontaneous emphysema. J. Toxicol. Environ. Health.

[62] Pourfathi, M., S. Kadlecek, Y. Xin, S. Siddiqui, M. Cereda, H. Profka, H. Hamedani, and R. R. Rizi. In vivo-quantitative assessment of lung injury using carbon-13 HP-MRI in a two-hit model of acid aspiration and VILI. In: D28. NOVEL IMAGING METHODS TO STUDY LUNG DISEASE. New York: American Thoracic Society, 2016, pp. A6620–A6620.

[63] Protti A, Cressoni M, Santini A, Langer T, Mietto C, Febres D, Chierichetti M, Coppola S, Conte G, Gatti S, Leopardi O, Masson S, Lombardi L, Lazzerini M, Rampoldi E, Cadringher P, Gattinoni L (2011). Lung stress and strain during mechanical ventilation: any safe threshold?. Am. J. Respir. Crit. Care Med..

[64] Quiros KAM, Nelson TM, Sattari S, Mariano CA, Ulu A, Dominguez EC, Nordgren TM, Eskandari M (2022). Mouse lung mechanical properties under varying inflation volumes and cycling frequencies. Sci. Rep..

[65] Raymondos K, Molitoris U, Capewell M, Sander B, Dieck T, Ahrens J, Weilbach C, Knitsch W, Corrado A (2012). Negative- versus positive-pressure ventilation in intubated patients with acute respiratory distress syndrome. Crit. Care.

[66] Rocco PRM, Souza AB, Faffe DS, Pássaro CP, Santos FB, Negri EM, Lima JGM, Contador RS, Capelozzi VL, Zin WA (2003). Effect of corticosteroid on lung parenchyma remodeling at an early phase of acute lung injury. Am. J. Respir. Crit. Care Med..

[67] Rohrs EC, Bassi TG, Fernandez KC, Ornowska M, Nicholas M, Wittmann JC, Reynolds SC (2021). Diaphragm neurostimulation during mechanical ventilation reduces atelectasis and transpulmonary plateau pressure, preserving lung homogeneity and PaO2/FIO2. J. Appl. Physiol..

[68] Romero PV, Faffe DS, Cañete C (2011). Dynamic nonlinearity of lung tissue: frequency dependence and harmonic distortion. J. Appl. Physiol..

[69] Rubini A, Carniel EL (2014). A review of recent findings about stress-relaxation in the respiratory system tissues. Lung.

[70] Sattari S, Mariano CA, Vittalbabu S, Velazquez JV, Postma J, Horst C, Teh E, Nordgren TM, Eskandari M (2020). Introducing a custom-designed volume-pressure machine for novel measurements of whole lung organ viscoelasticity and direct comparisons between positive- and negative-pressure ventilation. Front. Bioeng. Biotechnol..

[71] Sattari S, Eskandari M (2020). Characterizing the viscoelasticity of extra- and intra-parenchymal lung bronchi. J. Mech. Behav. Biomed. Mater..

[72] Sattari S, Mariano CA, Eskandari M (2023). Biaxial mechanical properties of the bronchial tree: characterization of elasticity, extensibility, and energetics, including the effect of strain rate and preconditioning. Acta Biomater..

[73] Sattari S, Mariano CA, Kuschner WG, Taheri H, Bates JHT, Eskandari M (2022). Positive- and negative-pressure ventilation characterized by local and global pulmonary mechanics. Am. J. Respir. Crit. Care Med..

[74] Seah AS, Grant KA, Aliyeva M, Allen GB, Bates JHT (2011). Quantifying the roles of tidal volume and PEEP in the pathogenesis of ventilator-induced lung injury. Ann. Biomed. Eng..

[75] Shekerdemian LS, Schulze-Neick I, Redington AN, Bush A, Penny DJ (2000). Negative pressure ventilation as haemodynamic rescue following surgery for congenital heart disease. Intensive Care Med..

[76] Slutsky AS (1999). Lung injury caused by mechanical ventilation. Chest.

[77] Sly PD, Collins RA, Thamrin C, Turner DJ, Hantos Z (2003). Volume dependence of airway and tissue impedances in mice. J. Appl. Physiol..

[78] Stenqvist O, Odenstedt H, Lundin S (2008). Dynamic respiratory mechanics in acute lung injury/acute respiratory distress syndrome: research or clinical tool?. Curr. Opin. Crit. Care.

[79] Suki B, Bates JHT (2011). Lung tissue mechanics as an emergent phenomenon. J. Appl. Physiol..

[80] Takeuchi M, Sedeek KA, Schettino GPP, Suchodolski K, Kacmarek RM (2001). Peak pressure during volume history and pressure–volume curve measurement affects analysis. Am. J. Respir. Crit. Care Med..

[81] Tarczy-Hornoch P, Hildebrandt J, Mates EA, Standaert TA, Lamm WJ, Hodson WA, Jackson JC (1996). Effects of exogenous surfactant on lung pressure–volume characteristics during liquid ventilation. J. Appl. Physiol..

[82] Eskandari, M., Sattari, S., & Quiros, K. The Role of Interspecies Variability on Positive- Versus Negativepressure Ventilation Mechanics. In C72. HOUSE OF ARDS... AND MECHANICAL VENTILATORY SUPPORT. American Thoracic Society, 2023, pp. A5776–A5776.

[83] Thomson A (1997). The role of negative pressure ventilation. Arch. Dis. Child..

[84] Voituron N, Zanella S, Menuet C, Lajard AM, Dutschmann M, Hilaire G (2010). Early abnormalities of post-sigh breathing in a mouse model of Rett syndrome. Respir. Physiol. Neurobiol..

[85] Wilson TA (1981). Mechanics of the pressure–volume curve of the lung. Ann. Biomed. Eng..

[86] Zosky GR, Janosi TZ, Adamicza A, Bozanich EM, Cannizzaro V, Larcombe AN, Turner DJ, Sly PD, Hantos Z (2008). The bimodal quasi-static and dynamic elastance of the murine lung. J. Appl. Physiol..

